# Clinical Features of Children with Retinoblastoma and Neuroblastoma

**DOI:** 10.1155/2020/9315784

**Published:** 2020-07-10

**Authors:** Xiaolian Fang, Huanmin Wang, Xiaoli Ma, Yongli Guo, Wei Yang, Shoulong Hu, Yue Qiu, Junyang Zhao, Xin Ni

**Affiliations:** ^1^Department of Otolaryngology Head and Neck Surgery, Beijing Children's Hospital, Capital Medical University, National Center for Children's Health, Beijing, China; ^2^Department of Surgical Oncology, Beijing Children's Hospital, Capital Medical University, National Center for Children's Health, Beijing, China; ^3^Beijing Key Laboratory of Pediatric Hematology Oncology, National Discipline of Pediatrics, Ministry of Education, MOE Key Laboratory of Major Diseases in Children, Hematology Oncology Center, Beijing Children's Hospital, Capital Medical University, National Center for Children's Health, Beijing, China; ^4^Beijing Key Laboratory for Pediatric Diseases of Otolaryngology Head and Neck Surgery MOE Key Laboratory of Major Diseases in Children, Beijing Pediatric Research Institute, Beijing Children's Hospital, Capital Medical University, National Center for Children's Health, Beijing, China; ^5^Department of Ophthalmology, Beijing Children's Hospital, Capital Medical University, National Center for Children's Health, Beijing, China; ^6^Department of Pediatric Oncology Center, Beijing Children's Hospital, Capital Medical University, National Center for Children's Health, Beijing, China

## Abstract

**Purpose:**

Retinoblastoma and neuroblastoma are the most common malignant extracranial solid tumors in children. This study aimed to summarize the clinical features, especially the delayed diagnosis in children with retinoblastoma and neuroblastoma.

**Methods:**

In a single hospital-based case-control study, a retrospective cohort of 175 children with retinoblastoma and neuroblastoma diagnosed from January 2016 to January 2018 were reviewed. The state of enucleation in retinoblastomas and pathological prognosis in neuroblastomas were outcome indicators. Hereby, the patients were divided into two groups, and clinical features including age at presentation and delayed diagnosis were compared.

**Results:**

A total of 112 patients with retinoblastoma and 63 with neuroblastoma were included. In the retinoblastoma cohort, the median age at presentation was 17.2 months (0.3–110 months). The mean delay of diagnosis was 1.6 ± 2.3 months, and the rate of enucleation was 61.6%. Unilateral disease, the International Classification of Intraocular Retinoblastoma (IIRC) stage E, and delay of diagnosis over 2.5 months were independent risk factors of ocular outcomes. Notably, the risk of enucleation was increased by 474% when the delay was longer than 2.5 months. In the neuroblastoma cohort, the delay of diagnosis of the unfavorable histology (UH) group was longer than that of the favorable histology (FH) group (1.9 months vs. 1.4 months, *P*=.487). The levels of serum ferritin and neuron-specific enolase were higher in the UH group than in the FH group (*P* < .05).

**Conclusions:**

This study summarized the clinical features and diagnosis biomarkers of retinoblastoma and neuroblastoma patients in China. These results might help to focus on early detection and treatment in children with retinoblastoma and neuroblastoma.

## 1. Introduction

Retinoblastoma and neuroblastoma, which are more common in children than in adults, are different from adult-onset cancers in tumorigenesis and transformation [1,2]. They are both embryonal tumors that originate from developing organs and demonstrate a close biological connection with those organs [3–5]. Moreover, these tumors have an early age of onset and rapid progression [6]. According to the International Classification of Childhood Cancer (ICCC), neuroblastoma is classified as group IVa and retinoblastoma as group V [7]. In terms of molecular mechanisms of pathogenesis, mutation or amplification in specific genes, including retinoblastoma 1 (RB1) [8, 9], MYCN [10], TP53 [11], and DEAD box 1 [12], are found in both diseases.

Despite unprecedented progresses in multiple therapeutics in the past decades, enucleation and unfavorable prognosis are still challenges in patients with retinoblastoma and neuroblastoma [1]. There is no consensus on the relationship between the delayed diagnosis and adverse outcomes of these two diseases. Moreover, diagnostic biomarkers of retinoblastoma and neuroblastoma may contribute to early diagnosis and improve prognosis [13]. Therefore, we aimed to summarize the clinical characteristics and diagnostic biomarkers of patients with retinoblastoma and neuroblastoma from a tertiary hospital, the National Center of Children's Health.

## 2. Materials and Methods

### 2.1. Study Design

This retrospective, single-institution study was reviewed and approved by Beijing Children's Hospital, Capital Medical University Review Board (IRM2020-k-4). All children with retinoblastoma and neuroblastoma that presented to Beijing Children's Hospital from January 2016 to January 2018 were reviewed. Only newly diagnosed patients who had not received previous treatment were included. The age range was 0 to 18 years. All retinoblastoma and neuroblastoma patients were followed up for at least 2 years after diagnosis.

### 2.2. Data Collection

The medical records of patients with retinoblastoma and neuroblastoma were reviewed to identify clinical characteristics and gather a complete clinical history. Data concerning demographic characteristics, family history, onset of symptoms, time from presenting symptoms to diagnosis, age at presentation, laterality, treatment, outcomes, IIRC/INSS classification, and the levels of lactate dehydrogenase (LDH) in retinoblastoma patients and the levels of LDH, serum ferritin (SF), and neuron-specific enolase (NSE) in neuroblastoma patients were collected from medical records.

Enucleation is a major concern for retinoblastoma patients; therefore, we used enucleation as an outcome indicator. The patients were divided into the enucleation (ENU) group and the no enucleation (NENU) group. In the neuroblastoma cohort, the patients were divided into favorable histology (FH) and unfavorable histology (UH) groups based on pathological classification, onset age, tumor pathology type, degree of differentiation, mitosis-karyorrhexis index (MKI), and other factors [14]. The prognosis outcomes of the FH and UH groups were used as outcome indicators among neuroblastoma patients. Moreover, the expression of serum LDH in retinoblastoma was counted, and 110–295 U/L was the normal range in children.

### 2.3. Statistical Analysis

Data were analyzed using IBM SPSS software (version 20.0) and JMP software (version 14.0). Categorical data were described as numbers and percentages. Quantitative data were described as means ± standard deviations or medians with ranges. Comparisons of categorical variables between different groups were performed using the chi-square test. When more than 20% of the cells had an expected count less than 5, Monte Carlo correction was used. Odds ratios (ORs) with 95% confidence intervals (CIs) were calculated from logistic regression models. Parametric tests were used for data with a normal distribution, and nonparametric tests were used for data that were abnormally distributed. *P* < .05 was considered significant unless otherwise specified.

## 3. Results

### 3.1. Patient Characteristics

**Figure 1:**
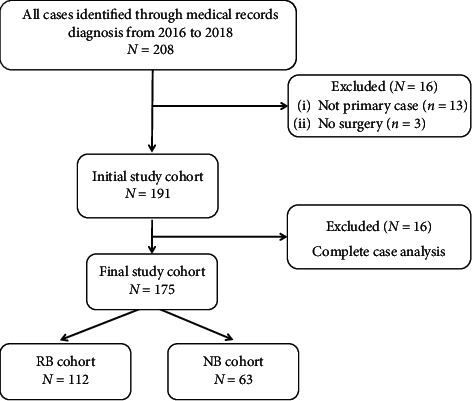
Flowchart illustrating subject enrollment.

**Figure 2:**
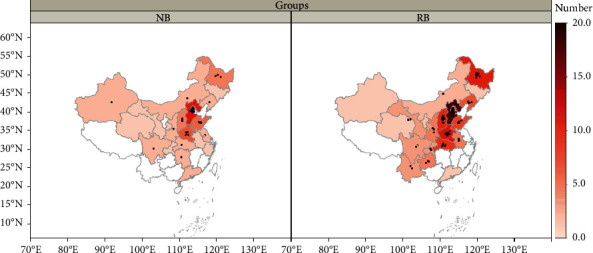
The residence distribution map of children with retinoblastoma and neuroblastoma patients in our cohort. The left shows the neuroblastoma (NB) cohort and the right shows the retinoblastoma (RB) cohort. The color depth indicates the population density. The black dots represent the number of population.

**Figure 3:**
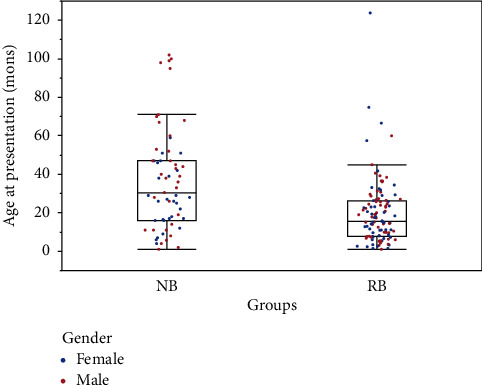
The age at presentation in neuroblastoma (NB) and retinoblastoma (RB) groups. The left shows the NB cohort, and the right shows the RB cohort; mons = months.

The medical records of 208 patients were reviewed. One hundred and seventy-five patients were eligible: 112 patients with retinoblastoma and 63 patients with neuroblastoma (Figure 1). Patients were scattered geographically, representing 23 provinces and three municipalities, four economic regions, and seven ethnic groups; patients were from both rural and urban areas (Figure 2). In the retinoblastoma cohort, the median age at presentation was 17.2 months (range: 0.3–110 months). Approximately half of the retinoblastoma patients were males (57/112, 50.9%), and the Han ethnicity accounted for 92.9% (104/112) of patients. In the neuroblastoma cohort, the median age at presentation was 30.5 months (range: 1.0–102.0 months). The male : female ratio was 1.25, and there were 35 male and 28 female patients. The Han ethnicity accounted for 96.8% (61/63) of patients (Figure 3 and Table 1).

### 3.2. Main Characteristics of Patients with Retinoblastoma

#### 3.2.1. Clinical Features of the Retinoblastoma Cohort

Overall, 88 (78.6%) of 112 retinoblastoma patients presented at younger than 2 years of age, and 109 patients (97.3%) were younger than 5 years of age at presentation. The most common symptoms of retinoblastoma patients were leukocoria (64/112, 57.1%) and strabismus (18/112, 16.1%). Over half of the cases were unilateral retinoblastoma (72/112, 64.3%). The mean time from presenting symptoms to diagnosis was 1.6 ± 2.3 months (median: 0.8 months; range: 0.12 to 12.0 months). The delay of diagnosis was 1.7 months in patients with unilateral disease and 1.3 months in patients with bilateral disease (*P*=.502). A delay longer than 2 months occurred in 18 patients (16.1%), and a delay over 6 months occurred in 5 (4.5%) patients.

Of 112 patients with retinoblastoma, 69 (61.6%) underwent enucleation before or after chemotherapy. Univariate analysis showed that unilateral disease (*P*=.004), delayed diagnosis longer than 2.5 months (*P*=.039), stage E (*P*=.003), and red and inflamed eyes (*P*=.015) were prognostic factors of ocular outcomes (Table 2). Multivariate analyses showed that unilateral disease, IIRC stage E, and delay of diagnosis over 2.5 months were independent risk factors of ocular outcomes when factoring in other covariates. Notably, the risk of enucleation was increased by 474% when the delay of diagnosis was longer than 2.5 months (Table 3).

#### 3.2.2. The Level of Serum LDH in Retinoblastoma

Among 119 patients with retinoblastoma, LDH levels were assessed in 110. Forty-two patients had elevated LDH, with an abnormal rate of 55.5% (61/110). The abnormal rates of LDH were 37.7% (20/53) in the ENU group and 71.9% (41/57) in the NENU group.

### 3.3. Main Characteristics of Neuroblastoma Patients

#### 3.3.1. Clinical Features of the Neuroblastoma Cohort

The main primary sites of neuroblastomas were the abdomen (56/63, 88.9%), retroperitoneum (29/63, 46.0%), and adrenal gland (24/63, 38.1%).  Table 4 shows the histopathological features of the tumors, including neuroblastomas, ganglioneuroblastomas (GNBs), and ganglioneuromas (GNs). The most common symptoms in neuroblastoma patients were an enlarged mass (30/63, 47.6%) and abdominal pain (14/63, 22.2%).

**Figure 4:**
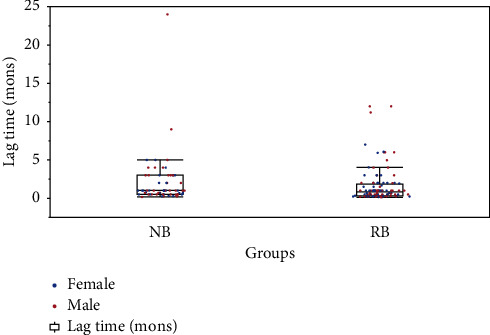
The lag time from presentation to diagnosis in neuroblastoma (NB) and retinoblastoma (RB) groups. The left shows the NB cohort, and the right shows the RB cohort; mons = months.

The median delay of diagnosis was 1.0 months (range: 0.2 to 24.0 months) in patients with neuroblastoma (Figure 4). Among these, 82.5% (52/63) had a delay longer than 2 weeks, 22.2% (14/63) had a delay longer than 2 months, and 1 patient had a delay longer than 6 months. There were 27 patients in the FH group and 36 in the UH group. The delay of diagnosis in the UH group was longer than that in the FH group (1.9 months vs. 1.4 months, *P*=.487). Univariate analysis showed that male gender (*P*=.04) and advanced age at presentation (*P* < .001) were prognostic factors.

#### 3.3.2. Biomarkers in Neuroblastoma Patients

SF was higher in the UH group than in the FH group (718.3 ng/mL vs. 208.2 ng/mL, *P*=.044). NSE was also higher in the UH group than in the FH group (247.8 ng/mL vs. 146.7 ng/mL, *P*=.028). In addition, the abnormal rate of LDH in the UH group was 95.5%, which was higher than that observed in the FH group (61.5%; *P*=.01).

## 4. Discussion

Retinoblastoma and neuroblastoma are the most common malignant extracranial solid tumors in children. These cancers increased the burden on the state and caused pain in children and anxiety in parents [6, 15]. Our findings suggested that delayed diagnosis may affect the prognosis of pediatric retinoblastoma and neuroblastoma patients, inevitably leading to heavier economic and psychological burdens. Although the survivals of retinoblastoma and neuroblastoma have improved significantly, early diagnosis is still closely correlated with better prognosis, earlier disease stage, and smaller tumor size [16]. Pediatric solid tumors have unique anatomical locations, cellular origins, and clinical presentations [2]. As pediatric embryonal tumors, retinoblastoma and neuroblastoma share similar histogenesis and histopathological features, despite having different anatomical locations [4, 17]. No study has examined the clinical features or delayed diagnosis in both diseases. Therefore, this study aimed to describe the clinical features and diagnostic biomarkers of these two diseases to identify clinical features that affect the prognosis.

Because of the retrospective nature of the study and the short follow-up time, we selected ocular outcome and histology as short-term outcomes. Although these are not as accurate as survival analysis, they are still representative. Eye preservation is a concerning indicator for retinoblastoma patients and their parents, and the FH and UH classification can correctly predict prognosis in neuroblastomas according to the International Neuroblastoma Pathology Classification System [14, 18].

In the retinoblastoma cohort, delayed diagnosis longer than 2.5 months was an adverse prognostic factor, which is in agreement with the results of other studies [16, 19]. Xiao et al. [19] found that high-risk retinoblastoma had a longer interval from symptom presentation to enucleation than standard-risk retinoblastoma. According to Soliman et al. [16], delayed diagnosis is significantly associated with advanced disease in both unilateral and bilateral retinoblastomas. Moreover, children with a delay of diagnosis longer than 30 days are at a significantly higher risk of extraocular invasion [19]. Early diagnosis in retinoblastoma is a key concern. In the neuroblastoma cohort, delayed diagnosis interval of the UH group was longer than that of the FH group, but it was not statistically significant. However, it is controversial that the real revenue for early screening in neuroblastoma patients considering the clinical heterogeneity of the disease. In 1985, a nationwide large-scale screening program for NB was started in Japan, but it was stopped in 2003 because of suspected overdiagnosis [20, 21]. Therefore, more randomized controlled trials and careful consideration are needed when implementing population-wide strategies.

Our study also found that patients with unilateral retinoblastoma typically presented later than patients with bilateral disease, and they had a worse prognosis. Patients with bilateral retinoblastoma are more likely to have a family history and experience early onset, multifocal tumors, and more severe symptoms [5]. This might be related to the timely detection of bilateral retinoblastomas by standard surveillance [22]. This also highlights the importance of early diagnosis of retinoblastomas. The causes of delayed diagnosis of retinoblastoma patients are mainly patient delay and healthcare system reasons [23, 24]. For patients, ignoring symptoms and delaying seeking medical care are the most common reasons for delayed diagnosis [25]. Delayed diagnosis after seeing a general physician or nonspecific presentation is also common [26]. Moreover, socioeconomic factors, forcing some parents to refuse to face the problems after enucleation, also lead to poor compliance [17]. An example is that the age at diagnosis was significantly older in children from lower socioeconomic provinces in Argentina [27].

The recent development of body fluid-based biomarker analysis has drawn great interest. This technique overcomes the limitations of traditional tissue-based tumor analysis [13]. For neuroblastoma, the classic urine catecholamine or catecholamine metabolites, LDH, SF, and NSE are widely used in clinical disease diagnosis and prognosis [15, 28]. In our study, the levels of LDH, NSE, and SF were higher in the UH group than in the FH group, which is consistent with the result of previous studies [29–34]. LDH is an important enzyme in the glycolytic pathway in tumor tissues and can be used to evaluate tumor burden and prognosis [29]. NSE, which is closely related to the advanced disease stage and poor prognosis, is a cell-specific isozyme of glycolytic enolase found in neurons, peripheral nerve tissues, and neurosecretory tissues [30]. Massaron et al. [35] found that repeated NSE measurements during follow-up could predict recurrence in patients without any clinical symptoms. There are two forms of SF: the glycosylated form that the cell actively secretes and the nonglycosylated form, which is directly released by damaged cells [31]. Moreover, the level of ferritin returns to normal with clinical remission, suggesting that the tumor is the source of elevated ferritin [33, 34]. Additionally, several genetic alterations including MEG3 polymorphisms [36], ERCC1/XPF [37], and NRAS [38] were associated with the occurrence of neuroblastoma and might serve as important diagnosis biomarkers in the future [13]. However, few clinical biomarkers are used in retinoblastoma, and we explored the value of LDH in disease diagnosis but without significance. More accurate biomarkers for therapeutic stratification and prognostication are needed for retinoblastoma.

Our findings indicated an adverse effect of delayed diagnosis on the outcomes of patients with retinoblastoma and neuroblastoma. This should be considered when formulating or revising national health policies. The current situation of delayed diagnosis of retinoblastoma and neuroblastoma emphasizes the necessity of popularization, dissemination, and promotion of medical science. Additionally, multidisciplinary management and algorithms must be widely used to reduce diagnosis and treatment delays by general practitioners.

There are several limitations in our study because of its retrospective nature. This was a single-center study, and the reliability of our conclusions would increase by cooperation with other centers. Additionally, we could not obtain specific detailed information on the delay of diagnosis. Therefore, we did not analyze the differences between parents' delays and doctor's delays. Finally, only short-term follow-up was performed, which reduces the reliability of the results. We will continue to follow our study cohort and provide results in larger cohorts. More detailed information and reliable evidence are needed.

## 5. Conclusion

This study summarized the clinical features and diagnostic biomarkers of retinoblastoma and neuroblastoma in China. These results might help to focus on the early detection and treatment of children with retinoblastoma and neuroblastoma.

## Figures and Tables

**Table 1:** Demographic characteristic of retinoblastoma and neuroblastoma in the study cohort.

Variables	Retinoblastoma		Neuroblastoma	
	*N* (112)	(%)	*N* (63)	(%)
Sex				
Male	57	50.9	35	55.6
Female	55	49.1	28	44.4

Ethnicity group				
Han	104	92.9	61	96.8
Non-Han	8	7.1	2	2
Age at presentation (median, mons)	13.7 (0. 3–110.0)		30.5 (1–102)	

Main complaints				
Leukocoria/mass	64	57.1	30	47.62
Strabismus/abdominal pain	18	161	14	22.22
Lag time (mean, mons)	1.6 ± 2.3		0.9 (0.2–24.1)	

Outcomes				
No enucleation/favorable	43	38.4	27	42.9
Enucleation/unfavorable	69	61.6	36	57.1

mons = months; *N* = number.

**Table 2:** Children with retinoblastoma categorized by ocular outcomes.

Variable	ENU (*n* = 34)	NENU (*n* = 36)	*P* value
Sex			
Female	34	21	0.964
Male	35	22	

Age at presentation (median, mons)	14.0 (0.8–72.0)	12.5 (0.3–110.0)	

Ethnicity			
Han	64	40	0.957
Non-Han	5	3	

Residence			
Rural	28	23	0.182
Urban	41	20	

Lateral			
Unilateral	53	19	**<0.001** ^*∗*^
Bilateral	16	24	

Presentation			
Leukocoria/others	40/29	24/19	0.822
Strabismus/others	8/61	10/33	0.102
Red and inflamed/others	12/57	1/42	**0.015** ^*∗*^

IIRC			
Stage D	22	26	**0.003** ^*∗*^
Stage E	47	17	

Lag time (mons)	1.9 ± 2.7	1.1 ± 1.2	0.075

Delayed diagnosis			
Lag time ≤ 2.5 mons	15	3	**0.039** ^*∗*^
Lag time > 2.5 mons	54	40	

ENU, enucleation group; NENU, no enucleation group; mons, months; ^*∗*^*P* < 0.05.

**Table 3:** Odds ratios of ocular outcomes for retinoblastoma by the multiple logistic regression model.

Variables	Odds ratio	95% CI	*P* value
Lateral			
Bilateral	1		
Unilateral	3.257	1.302–8.142	**0.012** ^*∗*^

Presentation			
Others	1		
Red and inflamed	5.773	0.660–50.542	0.113

IIRC			
D stage	1		
E stage	3.257	1.302–8.142	**0.012** ^*∗*^

Delayed diagnosis			
Lag time ≤2.5 mons	1		
Lag time >2.5 mons	5.740	1.335–24.688	**0.019** ^*∗*^

CI = confidence interval; mons = months. ^∗^ *P* < 0.05

**Table 4:** Histopathology features of neuroblastomas.

Variants	*N*
Histopathology and degree of differentiation	(*n* = 63)
NB	47 (71.21%)
Undifferentiated or poorly differentiated	24
Differentiating	20
Unknown	3
GNB	14 (22.22%)
Nodular	12
Mixed	2
GN	2 (3.03%)
Mature	2

NB = neuroblastoma; GNB = ganglioneuroblastoma; GN = ganglioneuroma.

## Data Availability

The datasets used and/or analyzed during the current study are available from the corresponding author on reasonable request. The data are not publicly available due to privacy and ethical restrictions.

## References

[1] Scotting P. J., Walker D. A., Perilongo G. (2005). Childhood solid tumours: a developmental disorder. *Nature Reviews Cancer*.

[2] Messahel B., Nash R., Jeffrey I., Pritchard-Jones K., Hing S. (2005). Clinical features of molecular pathology of solid tumours in childhood. *The Lancet Oncology*.

[3] Albert D. M., Plum L. A., Yang W. (2005). Responsiveness of human retinoblastoma and neuroblastoma models to a non-calcemic 19-nor Vitamin D analog. *Journal of Steroid Biochemistry Molecular Biology*.

[4] Roelofs K., Shaikh F., Astle W., Gallie B. L., Soliman S. E. (2018). Incidental neuroblastoma with bilateral retinoblastoma: what are the chances?. *Ophthalmic Genetics*.

[5] Mendoza P. R., Grossniklaus H. E. (2015). The biology of retinoblastoma. *Progress in Molecular Biology and Translational Science*.

[6] Dimaras H., Corson T. W., Cobrinik D. (2015). *Nature Reviews. Disease Primers*.

[7] Steliarova-Foucher E., Stiller C., Lacour B., Kaatsch P. (2005). International Classification of Childhood Cancer, third edition. *Cancer*.

[8] Knudson A. G., Strong L. C. (1972). , Mutation and cancer: neuroblastoma and pheochromocytoma. *American Journal of Human Genetics*.

[9] Maris J. M., Knudson A. G. (2015). Revisiting tissue specificity of germline cancer predisposing mutations. *Nature Reviews Cancer*.

[10] Brodeur G. M., Saylors R. L. (1991). Neuroblastoma, retinoblastoma, and brain tumors in children. *Current Opinion in Oncology*.

[11] Oh L., Hafsi H., Hainaut P., Ariffin H. (2019). p53, stem cell biology and childhood blastomas. *Current Opinion in Oncology*.

[12] Godbout R., Li L., Liu R.-Z., Roy K. (2007). Role of DEAD box 1 in retinoblastoma and neuroblastoma. *Future Oncology*.

[13] Trigg R. M., Shaw J. A., Turner S. D. (2019). Opportunities and challenges of circulating biomarkers in neuroblastoma. *Open Biology*.

[14] Shimada H., Umehara S., Monobe Y. (2001). International neuroblastoma pathology classification for prognostic evaluation of patients with peripheral neuroblastic tumors: a report from the children’s. *Cancer Group*.

[15] Colon N. C., Chung D. H. (2011). Neuroblastoma. *Advances in Pediatrics*.

[16] Soliman S. E., Eldomiaty W., Goweida M. B., Dowidar A. (2017). Clinical presentation of retinoblastoma in Alexandria: a step toward earlier diagnosis. *Saudi Journal of Ophthalmology*.

[17] Butros L. J., Abramson D. H., Dunkel I. J. (2002). Delayed diagnosis of retinoblastoma: analysis of degree. *Cause, and Potential Consequences*.

[18] Brodeur G. M., Pritchard J., Berthold F. (1993). Revisions of the International Criteria for Neuroblastoma Diagnosis, Staging, and Response to Treatment. *Journal of Clinical Oncology*.

[19] Xiao W., Ye H., Zeng H. (2019). Associations among socioeconomic factors, lag time, and high-risk histopathologic features in eyes primarily enucleated for retinoblastoma. *Current Eye Research*.

[20] Shinagawa T., Kitamura T., Katanoda K., Matsuda T., Ito Y., Sobue T. (2017). The incidence and mortality rates of neuroblastoma cases before and after the cessation of the mass screening program in Japan: a descriptive study. *International Journal of Cancer*.

[21] Katanoda K. (2016). Neuroblastoma mass Screening&mdash;What can we learn from it?. *Journal of Epidemiology*.

[22] Kamihara J., Bourdeaut F., Foulkes W. D. (2017). Retinoblastoma and neuroblastoma predisposition and surveillance. *Clinical Cancer Research*.

[23] Dang-Tan T., Franco E. L. (2007). Diagnosis delays in childhood cancer. *Cancer*.

[24] Araz N. C., Guler E. (2015). Delays in diagnosis of childhood cancer in southeastern Turkey and the associated factors. *Pediatric Hematology and Oncology*.

[25] Dimaras H., Corson T. W. (2019). Retinoblastoma, the visible CNS tumor: a review. *Journal of Neuroscience Research*.

[26] Ramirez-Ortiz M. A., Ponce-Castaneda M. V., Cabrera-Munoz M. L., Medina-Sanson A., Liu X., Orjuela M. A. (2014). Diagnostic delay and sociodemographic predictors of stage at diagnosis and mortality in unilateral and bilateral retinoblastoma. *Cancer Epidemiology Biomarkers & Prevention*.

[27] Moreno F., Sinaki B., Fandiño A., Dussel V., Orellana L., Chantada G. (2014). A population-based study of retinoblastoma incidence and survival in Argentine children. *Pediatric Blood & Cancer*.

[28] Matthay K. K., Maris J. M., Schleiermacher G. (2016). Neuroblastoma. *Nature Reviews. Disease Primers*.

[29] Pang Q. M., Li K., Ma L. J., Sun R. P. (2015). Clinical research on neuroblastoma based on serum lactate dehydrogenase. *Journal of Biological Regulators and Homeostaic Agents*.

[30] Georgantzi K., Sköldenberg E. G., Stridsberg M. (2018). Chromogranin A and neuron-specific enolase in neuroblastoma: correlation to stage and prognostic factors. *Pediatric Hematology and Oncology*.

[31] Isgrò M. A., Bottoni P., Scatena R. (2015). Neuron-specific enolase as a biomarker: biochemical and clinical aspects. *Advances in Cancer Biomarkers*.

[32] Parodi S., Perfumo C., Garaventa A. (2010). MDM2 SNP309 genotype is associated with ferritin and LDH serum levels in children with stage 4 neuroblastoma. *Pediatric Blood & Cancer*.

[33] Hann H. W., Stahlhut M. W., Evans A. E. (1986). Source of increased ferritin in neuroblastoma: studies with concanavalin A-sepharose binding. *Journal of the National Cancer Institute*.

[34] Hann H. W., Evans A. E., Siegel S. E. (1985). Prognostic importance of serum ferritin in patients with Stages III and IV neuroblastoma: the Childrens Cancer Study Group experience. *Cancer Research*.

[35] Massaron S., Seregni E., Luksch R. (1998). Neuron-specific enolase evaluation in patients with neuroblastoma. *Tumor Biology*.

[36] Zhuo Z.-J., Zhang R., Zhang J. (2018). MEG3 associations between lncrna polymorphisms and neuroblastoma risk in chinese children. *Aging*.

[37] Zhuo Z.-J., Liu W., Zhang J. (2018). Functional polymorphisms at ERCC1/XPF genes confer neuroblastoma risk in Chinese Children. *EBioMedicine*.

[38] Li S., Zhuo Z., Chang X. (2020). NRAS Rs2273267 A>T Polymorphism Reduces Neuroblastoma Risk in Chinese Children. *Gene*.

